# Pathogenicity and host-interacting mechanisms of enterogenic *Enterobacter cancerogenus* in silkworm

**DOI:** 10.3389/fmicb.2025.1548808

**Published:** 2025-03-26

**Authors:** Meng Luo, Linhui Lai, Zailin Wu, Xiaoli Ren, Jiacheng Zhao, Hongmei Liu, Yaohang Long

**Affiliations:** ^1^Biochemistry Teaching and Research Section, School of Basic Medical Sciences, Guizhou Medical University, Anshun, China; ^2^School of Public Health, The Key Laboratory of Environmental Pollution Monitoring and Disease Control, Ministry of Education, Guizhou Medical University, Anshun, China; ^3^Engineering Research Center of Medical Biotechnology, School of Biology and Engineering, Guizhou Medical University, Anshun, China; ^4^Key Laboratory of Biology and Medical Engineering, Immune Cells and Antibody Engineering Research Center of Guizhou Province, Guizhou Medical University, Anshun, China; ^5^Engineering Research Center of Health Medicine Biotechnology of Institution of Higher Education of Guizhou Province, Guizhou Medical University, Anshun, China

**Keywords:** *Enterobacter cancerogenus*, *Bombyx mori*, PM, intestinal microorganisms, transcriptomic analysis, interaction between *E. cancerogenus* and silkworms

## Abstract

**Introduction:**

*Enterobacter cancerogenus* (*E. cancerogenus*) is a facultative anaerobic, gram-negative bacterium that can be utilized for the biological control of pests. However, the molecular mechanisms underlying the pathogenicity of E. cancerogenus in insect hosts remain largely unexplored.

**Methods:**

In this study, the *Bombyx mori* model was employed to investigate the pathogenicity of *E. cancerogenus* strain ECL7, a bacterium pathogenic to silkworms, through whole-genome sequencing, 16S rDNA sequencing, and transcriptome analysis.

**Results:**

The results revealed that ECL7 harbors virulence genes associated with biofilm formation, adhesion, type III secretion system (T3SS), type VI secretion system (T6SS), and other factors, which collectively lead to damage to the peritrophic matrix (PM) and intestinal epithelial cells of the silkworm midgut following infection, and reduced silkworm larval survival rates and inhibited their growth and development. Additionally, ECL7 infection altered the composition and abundance of intestinal microorganisms, with *Enterobacteriaceae* and *Enterobacteriales* becoming dominant species. ECL7 also stimulated the expression of genes related to the Toll and IMD immune signaling pathways, resulting in the upregulation of antimicrobial peptide–related differentially expressed genes (DEGs). Furthermore, transcriptomic analysis revealed an upregulation of DEGs associated with oxidative stress in response to ECL7 infection.

**Discussion:**

This study provides valuable insights into the molecular mechanisms underlying the interaction between *E. cancerogenus* and silkworms. The findings contribute to the prevention and control of infections caused by this bacterium in sericulture production and offer novel ideas for the potential application of *E. cancerogenus* in pest biological control.

## 1 Introduction

The *Enterobacter cloacae* complex (ECC) comprises multiple species, including major members such as *Enterobacter cloacae* (*E. cloacae*), *Enterobacter hormaechei* (*E. hormaechei*), *Enterobacter roggenkampii* (*E. roggenkampii*), *Enterobacter kobei* (*E. kobei*), and *Enterobacter cancerogenus* (*E. cancerogenus*) (Liu et al., 2022; Mezzatesta et al., 2012; Ji et al., 2021). These species share highly similar physiological and biochemical characteristics (Chavda et al., 2016).

*E. cancerogenus*, a member of the ECC, is a facultative anaerobic, gram-negative bacterium known to cause nosocomial infections (Davin-Regli et al., 2019), including wound infections and sepsis (Hadano et al., 2018; Abbott and Janda, 1997). Researches have indicated that *E. cancerogenus* can cause the mortality in some pests. For example, it can lead to the death of the larvae of *Diprion pini* (Hymenoptera, Diprionidae), *Lutzomyia evansi* (Diptera, Psychodidae) and *Helicoverpa armigera* (Çelik and Sevim, 2022; Vivero et al., 2019; Pan et al., 2024). These findings indicate that *E. cancerogenus* not only acts as an insect pathogen but may also be applied in biological control strategies to manage agricultural pest populations. Additionally, certain ECC strains exhibit insecticidal activity; for example, *E. cloacae* SJ2 has been shown to cause mortality in mosquito larvae and termites (Harikrishnan et al., 2023). Research involving *Galleria mellonella* larvae has also explored the potential of *E. cloacae* and its insecticidal proteins in the biological prevention and control of these larvae (Zhu et al., 2024; Liao et al., 2023).

Lepidoptera is the second largest order in *Insecta*, encompassing many common pest and beneficial insect species. Most agricultural pests belong to the Lepidoptera order, such as *Helicoverpa armigera*, *Plutella xylostella*, *Spodoptera litura*, etc. (Pinto et al., 2017; Sang et al., 2016; Furlong et al., 2013). Meanwhile, the silkworm (*Bombyx mori* L.), a widely used lepidopteran model organism, is an economically important insect worldwide. The presence of pathogenic bacteria in its intestine can directly impact its industrial value (Meng et al., 2017). In 1998, Japanese scholar Kenji Watanabe isolated five *E. cloacae* strains from the intestines of silkworms; these bacteria carried plasmids capable of mediating horizontal gene transfer (Watanabe and Sato, 1998). However, in recent years, Li et al. demonstrated that the use of drugs such as phoxim pesticide, acetamiprid, and chlorantraniliprole to disrupt the intestinal peritrophic matrix (PM) of silkworms can significantly enhance the pathogenicity of exogenous *E. cloacae*, considerably increasing silkworm mortality (Li et al., 2020; Li et al., 2021; Zhu et al., 2023). The presence of such pathogens has caused substantial harm to the silkworm industry.

Bacterial diseases in silkworms can result from a combination of pathogenic and conditionally pathogenic bacteria. Pathogenic bacteria, such as *Bacillus bombyseptieus*, *Bacillus cereus*, and *Serratia* spp., have garnered extensive attention due to their strong pathogenicity (Huang et al., 2009; Li et al., 2019; Ishii et al., 2014). However, many conditionally pathogenic bacteria present in the intestines of silkworm larvae remain unexplored, and research on the pathogenicity of *E. cancerogenus* derived from silkworm intestines is particularly scarce. Although current studies have shown that *E. cancerogenus* can lead to the death of insect larvae, research on the molecular mechanisms underlying its interactions with insect hosts remains limited. In our previous study, we screened the intestines of infected silkworms and isolated *E. cancerogenus* strain ECL7, a member of the ECC. In this study, the pathogenicity of *E. cancerogenus* ECL7 in silkworms was investigated using genomic sequencing, 16S rDNA sequencing, and RNA-seq. These findings provide valuable insights for preventing infections caused by this bacterium during silkworm rearing and highlight its potential application in biological pest control.

## 2 Materials and methods

### 2.1 Bacterial culture

The ECL7 pathogen was isolated from the intestines of diseased silkworms and stored in our laboratory. The strain was streaked onto lysogeny broth (LB) agar and incubated at 37°C overnight. Single colonies were picked and cultured in LB liquid medium at 37°C with shaking at 200 rpm overnight.

### 2.2 ECL7 genome sequencing

Genomic DNA of ECL7 was extracted, and its concentration and quality were determined using a Nanodrop 2,500 instrument. Whole-genome sequencing, including both the complete genome and plasmids of ECL7, was performed using the Illumina and PacBio sequencing platforms (Shanghai Majorbio Bio-Pharm Technology Co., Ltd., Shanghai, China). Illumina’s high-accuracy and high-throughput data enable fine-scale gene annotation and species classification, while PacBio’s long-read data facilitate complete genome assembly and resolution of complex genomic structures. Local assembly and optimization of the assembly results were conducted using SOAPdenovo2,^1^ whereas complete chromosome and plasmid sequences were acquired using Unicycler (v 0.4.8). The phylogenetic status of ECL7 was analyzed using MEGA 6.0.^2^ The reads were assembled into contigs, and plasmid sequences were obtained from the bacterial genome assembly results using PlasFlow software.^3^ Plasmid annotation was performed using BLAST^4^ and the PLSDB database,^5^ followed by gene prediction using GeneMarkS software.^6^ The genomic coding sequences were predicted using Glimmer software.^7^ Finally, the virulence factors of ECL7 were predicted using the Virulence Factor Database (VFDB).^8^

### 2.3 Insect rearing

Fourth-instar silkworm larvae (Liang Guang 2 Hao) were reared on fresh mulberry leaves sterilized with a 75% ethanol solution. The larvae were maintained under a 12-h light/dark cycle at 25°C ± 1°C and 70% ± 5% relative humidity. The animal study was approved by GuiZhou Medical University the Animal Care Welfare Committee (NO.2400198).

### 2.4 Midgut infection of the silkworm and sample collection

ECL7 was incubated overnight at 37°C while being shaken at a speed of 200 rpm. Subsequently, the culture was centrifuged at 4500× g for 5 min. After that, the bacterial pellet was resuspended in sterile water. To obtain a bacterial suspension with a turbidity of 5 McFarland (5 MCF), the bacteria were counted using a bacterial turbidimeter (Qi Wei, Hangzhou, China). Finally, 5 mL of the 5 MCF bacterial suspension was concentrated to reach a bacterial count of 7.5 × 10^9^ CFU.

Fourth-instar silkworms of similar size were randomly categorized into two groups, each with three biological replicates (*n* = 50). Mulberry leaves were disinfected with 75% alcohol and subsequently rinsed three times with sterile water before the experiment. The mulberry leaves for the infected group were treated with an ECL7 bacterial suspension (7.5 × 10^9^ CFU/50 larvae), whereas the control group received the same volume of sterile water. The treated mulberry leaves were then fed to the fourth-instar larvae. Silkworms were observed daily, and mortality rates were recorded throughout the infection period.

The surface of each silkworm was wiped with a 75% ethanol solution and dissected under aseptic conditions. Midgut tissues and contents of 3 silkworms from both groups (control and ECL7-infected) were randomly collected for histological sectioning and gut microbial diversity analyses on the 1st, 2nd, and 4th days of ECL7 infection. Midgut tissues of 3 silkworms from both groups were also selected as individual samples for RNA-seq or quantitative real-time PCR (qRT-PCR) analysis on the 4th day of ECL7 infection. All samples, except those used for histopathological analysis, were stored at −80°C until further use.

### 2.5 Midgut histopathology

After the 1st, 2nd, and 4th days of infection, midgut tissues from both the control and ECL7 groups (*n* = 3) were randomly collected for histological sectioning. The midgut tissues were fixed in 4% paraformaldehyde for 24 h. Histological sections were prepared following standard protocols for dehydration and paraffin embedding (Pan et al., 2018). The sections were stained with hematoxylin and eosin, observed using a Nikon Eclipse E100 microscope (Nikon Microsystems, Tokyo, Japan), and photographed using a Nikon DS-U3 digital camera imaging system.

### 2.6 16S rDNA amplicon sequencing

After the 1st, 2nd, and 4th days of infection, midgut contents from silkworms in both the control and ECL7 groups (*n* = 3) were randomly collected for gut microbial diversity analyses. Genomic DNA was extracted from the midgut contents using the E.Z.N.A.™ Soil DNA Kit (Omega, Norcross, USA). Primer selection plays a pivotal role in determining the composition of the microbial community (He et al., 2023). For PCR amplification in this study, the widely – adopted universal primers 338F (ACTCCTACGGGAGGCAGCAG) and 806R (GGACTACHVGGGTWTCTAAT) were employed. Sequence libraries were constructed using the TruSeq™ DNA Sample Prep Kit (Illumina, San Diego, USA).

Sequencing was performed on the Illumina NextSeq 2000 platform (Illumina, San Diego, USA) at Shanghai Majorbio Bio-Pharm Technology Co., Ltd. (Shanghai, China). FLASH (version 1.2.7) was used to merge paired – end reads into single sequences, with quality control and filtering applied to both raw reads and merged sequences. Valid sequences were obtained by differentiating the samples based on the barcodes and primer sequences at the beginning and end of the sequences, followed by correcting the sequence orientation. The data were optimized using the DADA2 pipeline^9^ to denoise, remove erroneous sequences, obtain representative sequences, and extract abundance information for amplicon sequence variants (ASVs). QIIME2^10^ was used for clustering at the sample level and calculating variations in species abundance across samples. Additionally, R language tools were employed to construct pictures of communities at different taxonomic levels. ASVs with 97% similarity were selected and the alpha diversity indices were analyzed using Mothur (v1.30.2), *α*-diversity indices, including Chao1and Shannon indices. The microbial community composition and structure were examined using R and Python 3. Principal coordinates analysis (PCoA) was employed to assess similarities and differences between the microbial communities of the control and ECL7-treated groups, whereas *β*-diversity analysis was conducted to elucidate differences in community structure between the groups.

### 2.7 RNA-seq and data analysis

On the 4th day of infection, midgut tissues from the control and ECL7 groups (*n* = 3) were randomly selected and combined into one sample per group for RNA-seq analysis. Total RNA was extracted from the silkworm midgut using TRIzol reagent (Invitrogen, USA). The concentration and purity of the extracted RNA were assessed using a NanoDrop 2000 spectrophotometer (OD_260/280_ = 1.8–2.2), and RNA integrity was verified through 1% agarose gel electrophoresis. The transcriptomic library was constructed using the TruSeq™ RNA Sample Prep Kit (Illumina, USA), and sequencing was performed on the NovaSeq X Plus platform (Majorbio Bio-Pharm Technology Co., Ltd., Shanghai, China).

The raw sequencing data were subjected to quality control using Fastp (version 0.23.4), SeqPrep^11^ and Sickle^12^ to filter raw reads and obtain clean data. Reads with low-quality bases (Phred score < 20 at the 5′ end or Phred score < 3 at the 3′ end) or ambiguous bases (N content >5%) were discarded. All clean reads were aligned to the *B. mori* reference genome^13^ using TopHat2^14^ (Kim et al., 2013). The mapped reads were assembled and spliced using StringTie^15^ (Pertea et al., 2015). For comprehensive functional annotation of the transcriptome, the NR (v2023.07), Swiss-Prot (v2023.11), Pfam (v36.0), EggNOG (v2020.06), Gene Ontology (GO) (v2023.7), and Kyoto Encyclopedia of Genes and Genomes (KEGG) (v2023.09) databases were utilized. The expression levels of transcripts were quantified using RSEM software, employing the transcript per million quantitative index (Li and Dewey, 2011). Differentially expressed genes (DEGs) between groups were identified using DEGseq (v1.56.1) (Love et al., 2014). GO enrichment analysis of DEGs was performed using GOATOOLS,^16^ whereas KEGG enrichment analysis was conducted using R.

### 2.8 qRT-PCR analysis

qPCR was conducted to validate the results of transcriptome sequencing, with all real-time primers listed in Supplementary Table S1. After RNA extraction, 1 μg of RNA was reverse-transcribed into complementary DNA (cDNA) using the PrimeScript™ RT Master Mix (Takara, Tokyo, Japan). qRT-PCR was performed on the Applied Biosystems 7,500 System (Thermo, USA) using TB Green™ Premix Ex Taq™ (Takara, Tokyo, Japan). The total volume of the qPCR reactions and the program settings were consistent with those reported by Li et al. (2019). Each reaction was performed in triplicate. Data analysis was conducted using the 2^−ΔΔCT^ method, and expression levels were calculated as the log_2_ fold-change. The housekeeping gene *β-actin 3* was used as the internal reference gene.

### 2.9 Statistical analysis

In this study, date analyses were performed using the SPSS® 16.0 software (IBM, NY, USA) and the GraphPad Prism software, The results were analyzed by independent t-test and Kruskal – Wallis rank – sum test to determine the statistical significance of the difference between samples, and *p* < 0.05 indicated a significant difference.

## 3 Results

### 3.1 Identification of ECL7

**Figure 1:**
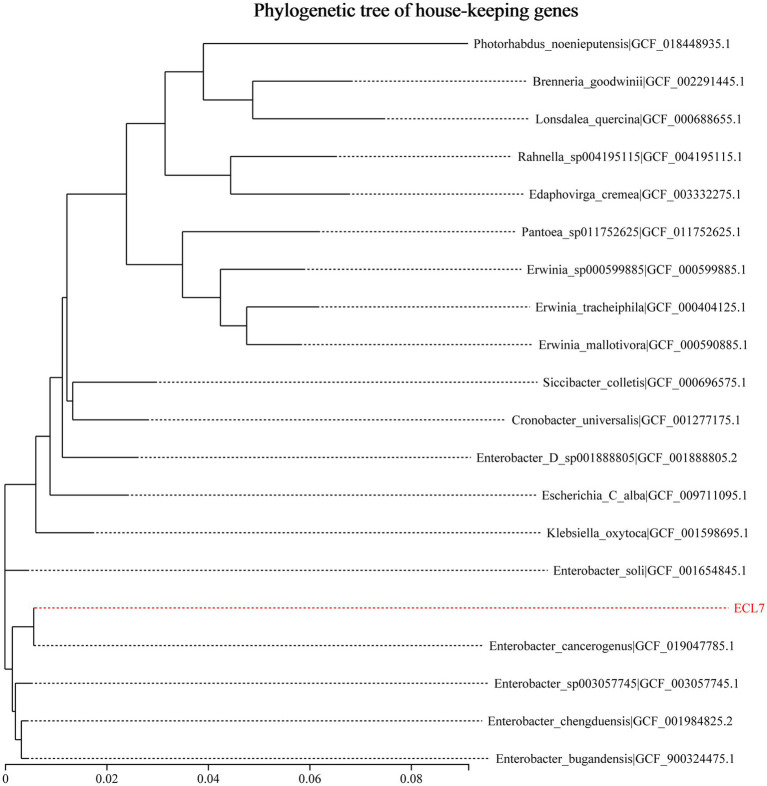
Phylogenetic analysis of *E. cancerogenus* strain ECL7 based on 31 housekeeping genes.

A phylogenetic analysis was performed using 31 housekeeping genes to identify the bacterial strain ECL7. Based on the generated phylogenetic tree, the 19 most closely related bacterial strains were selected at both the species and genus levels. As shown in Figure 1, ECL7 demonstrated a 99.5% sequence similarity to *E. cancerogenus* (GCF_019047785.1), with 81.3% genome coverage (Supplementary Table S2). Additionally, average nucleotide identity (ANI) analysis revealed 98.95% sequence identity between ECL7 and the type strain genome of *E. cancerogenus*, with 91.0% genome coverage.

### 3.2 Genomic analysis of ECL7

**Figure 2:**
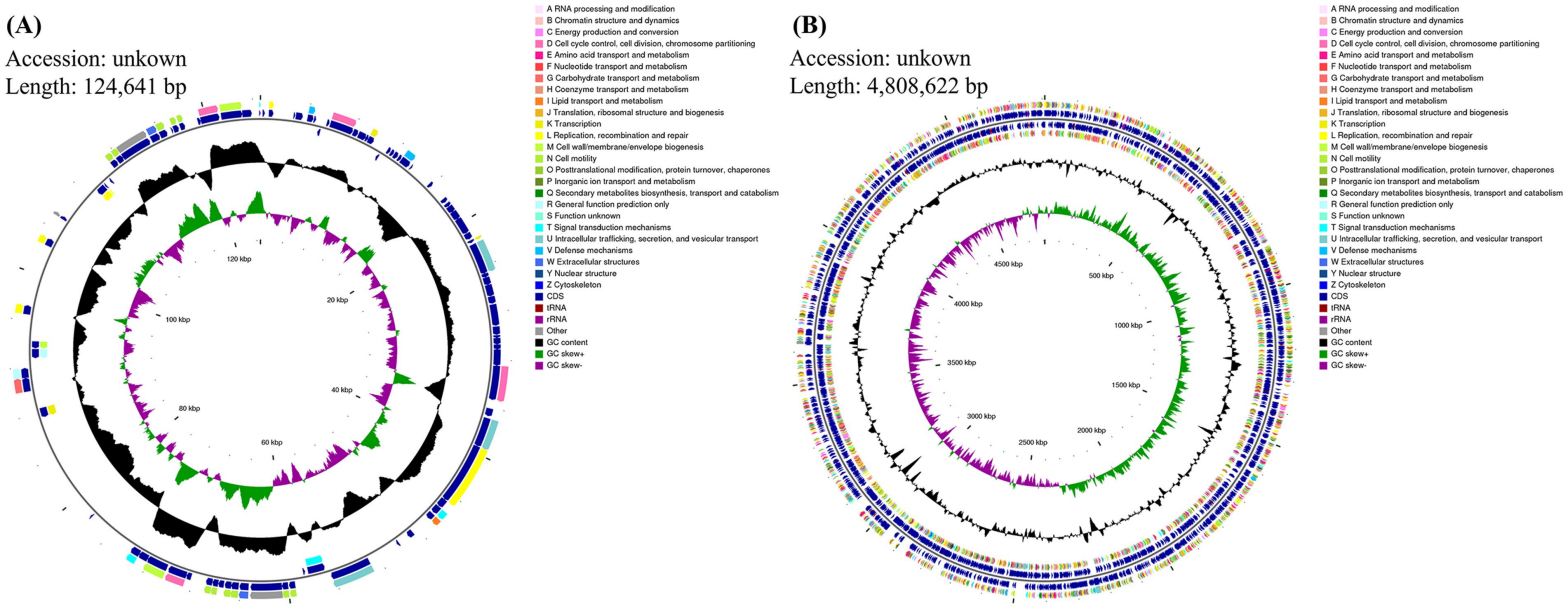
Whole-genome analysis of ECL7. **(A)** CGView circular map of the plasmid. **(B)** CGView circular map of the chromosome.

Whole-genome sequencing of ECL7 was performed using PacBio and Illumina sequencing platforms. The results revealed that ECL7 contains a single chromosome and one plasmid, designated as pC45-p2 (Figure 2). The genome coverage was 96.5%, with a total genomic length of 4,933,263 bp and a G + C content of 55.85%. The chromosome and plasmid lengths were 4,808,622 bp and 124,641 bp, respectively, with G + C contents of 55.84 and 56.34%. A total of 4,493 coding sequences were identified, corresponding to a gene-to-genome length ratio of 88.31%. Consequently, the proportion of intergenic regions within the genome was 11.69% (Table 1).

**Table 1:** General information on *Enterobacter cancerogenus* strain ECL7 genome.

Item	Description
Coding DNA Sequence (CDS)	4,493
Genome size (bp)	4,933,263
G + C content (%)	55.85
Number of scaffolds	96.5
Total gene length (bp)	4,356,588
Intergenic region length (bp)	576,675
Intergenic length/genome length (%)	11.69
Number of tRNA	87
Number of rRNA	25

### 3.3 Prediction analysis and detection of virulence factors

The VFDB predicted a total of 705 virulence genes for ECL7. Among these, 155 are for nutrition/metabolism, such as *iroD*, *entC*, and *iutA*; 110 for motility, including *cheD*, *flgE*, and *motA*; 107 for adherence, such as *ecpC*, *fimD*, and *htpB*; and 88 for immune modulation, including *gndA*, *ugd*, and *wcaJ*. Additionally, other virulence factor genes were associated with biofilm formation, stress survival, and exotoxin production. The analysis also identified 13 core genes (*tssA–M*) within the type VI secretion system (T6SS), which included flagellar and adhesion virulence genes, as well as other virulence-associated genes (Supplementary Table S3).

### 3.4 Characterization and histopathology of silkworm larvae after infection

**Figure 3:**
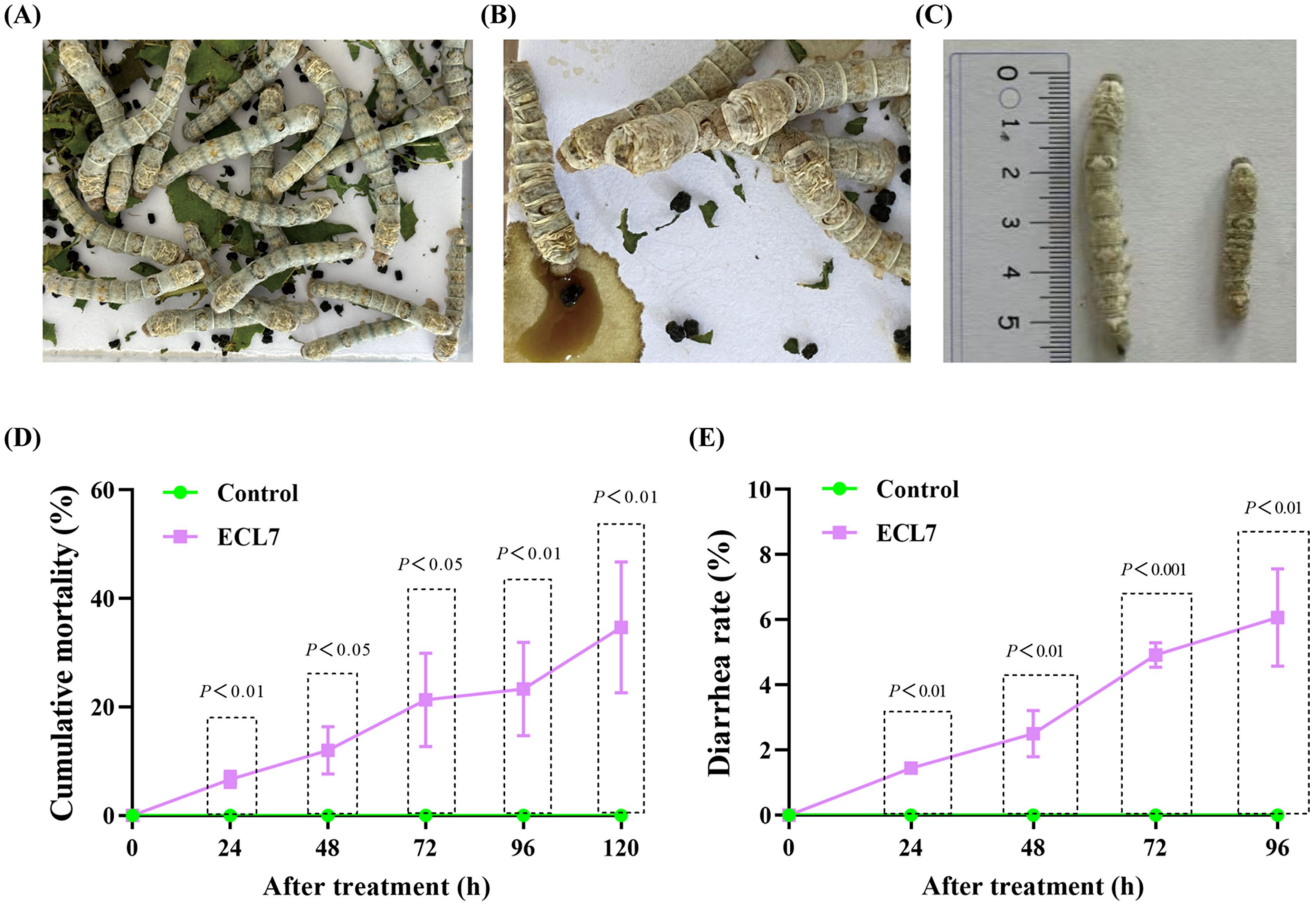
Effect of ECL7 on the growth and survival of silkworm larvae. **(A,B)** Phenotypic symptoms of silkworm larvae in control and ECL7-infected groups. **(C)** Body length of silkworm larvae. Scale bar = 1 cm. **(D)** The cumulative mortality rates of the control group and the ECL7-infected group from Day 1 (24 h) to Day 5 (120 h). **(E)** The diarrhea rate of the control group and the ECL7-infected group from Day 1 (24 h) to Day 4 (96 h). Error bars indicate the mean ± SD (*n* = 3).

Compared with the control group (Figure 3A), silkworms in the ECL7-treated group exhibited symptoms of intoxication, such as diarrhea and reduced growth rates (Figures 3B,C). The mortality rate of silkworms progressively increased in the ECL7-treated group and was significantly higher than that of the control group (*p* < 0.05). Specifically, on days 1 (24 h), 2 (48 h), 3 (72 h), and 4 (96 h), the mortality rates were 6.67, 12.00, 21.33, and 23.33%, respectively. On the 5th day (120 h), the mortality rate in the ECL7 – treated group was 34.67% due to bacterial effects (Figure 3D). This might be caused by the long – term accumulation of ECL7. In addition, the overall diarrhea rate caused by ECL7 infection in silkworms after 4 days was 14.94%, and the diarrhea rates of ECL7 – infected silkworm larvae on days 1–4 were 1.44, 2.50, 4.92, and 6.07% respectively, significantly higher than the control group (*p* < 0.05). These results show ECL7 infection affects silkworm health (Figure 3E).

**Figure 4:**
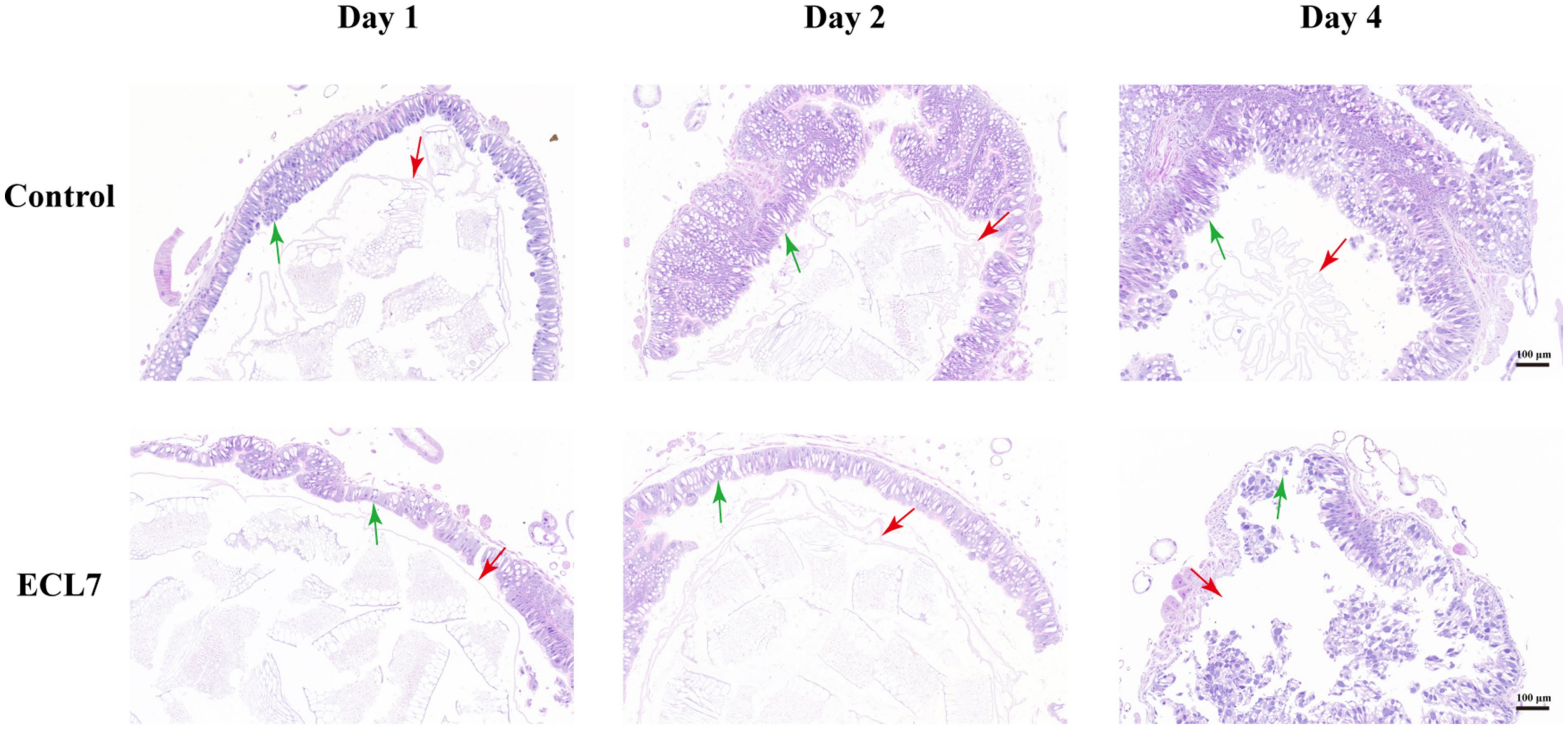
Morphological features of intestinal tissue after infection with ECL7 on the 1st, 2nd, and 4th days. Red and green arrows indicate the peritrophic matrix and epithelial cells, respectively. Scale bar = 100 μm.

Figure 4 illustrates the histological alterations in the control group and the ECL7-infected group on the 1st, 2nd, and 4th days. On Day 1 and Day 2, the intestinal epithelial cells in both the control and ECL7-infected groups remained intact. The peritrophic membrane (PM) presented as a continuous intact membranous structure, maintaining its normal morphological integrity. However, by day 4 in the ECL7-infected group, the intestinal epithelial cells suffered damage compared to the control group. The regular arrangement of the cells was disrupted, with cell nuclei pushing out of the damaged cells. Concurrently, the membranous structure of the PM became blurred, losing its continuity and showing signs of interruption and fragmentation. In conclusion, as the infection progressed, ECL7 caused damage to the intestinal epithelial cells and the PM structure by the fourth day.

### 3.5 *Bombyx mori* gut amplicon *α* and *β*-diversity indices

**Figure 5:**
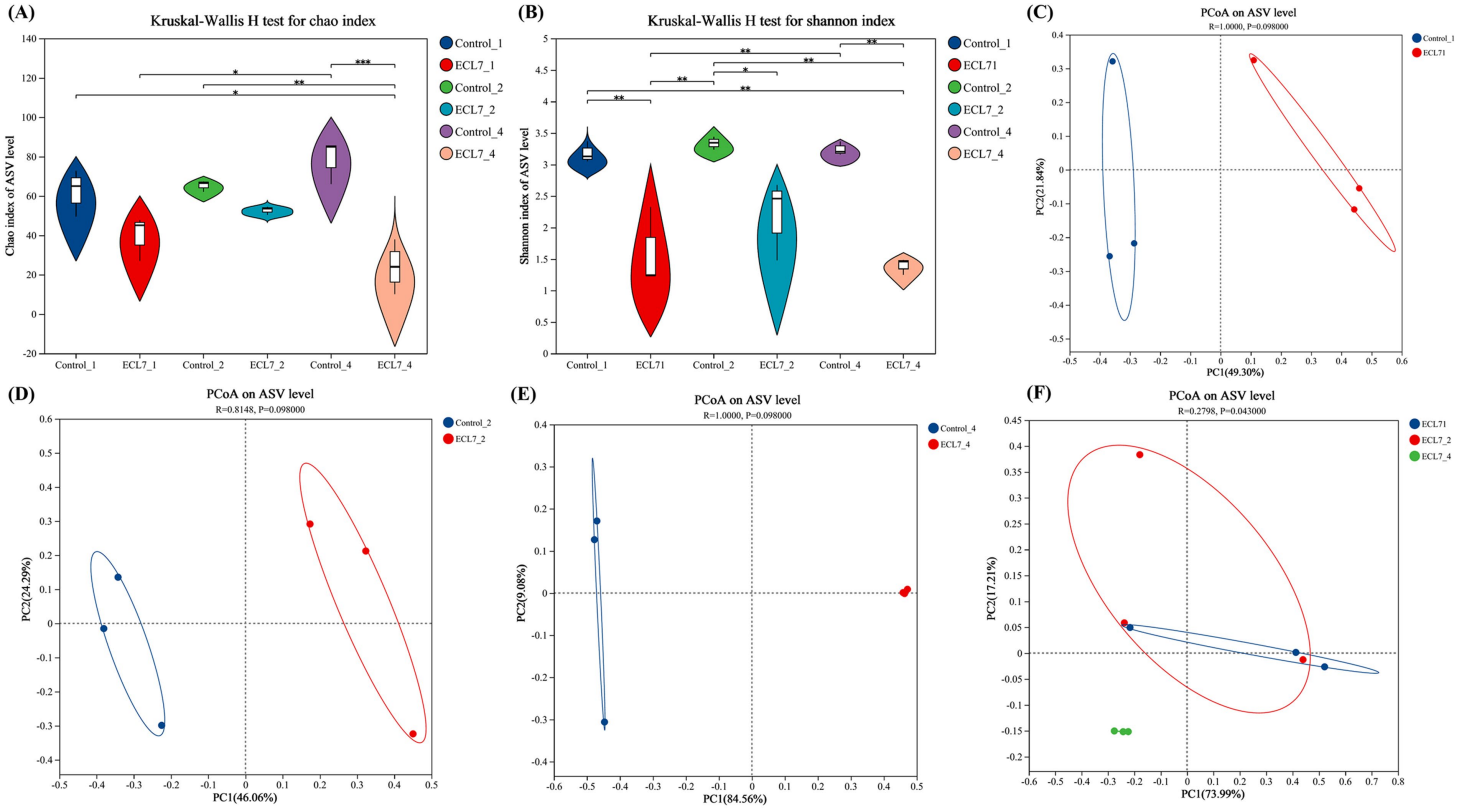
Effects of ECL7 infection on midgut microbial diversity indices. **(A,B)** Violin plots of microbial *α*-diversity analysis based on Chao1 and Shannon indices. **(C–E)** PCoA analysis showing separation of gut bacteria into treatment and control groups after ECL7 infection on days 1, 2, and 4, respectively. **(F)** PCoA analysis of the ECL7-treated group.

We employed 16S rDNA sequencing to investigate the microbial diversity in silkworms with and without ECL7 infection on the 1st, 2nd, and 4th days. Analysis of 18 samples yielded 947,322 optimized sequences. After denoising, 798,574 sequences were identified, with each sample containing between 39,203 and 49,679 sequences, and 1,421 ASVs were identified. Violin plots of microbial *α*-diversity indices, specifically Chao1 and Shannon, are shown in Figures 5A,B. The Chao1 index revealed no significant differences in microbial richness between silkworms with and without ECL7 infection during the first and second days, however, there was a significant difference in microbial richness between the silkworms in the control group and ECL7 group on the 4th day (*p* < 0.05). The Shannon index was significantly lower in the ECL7-infected group than in the control group on days 1, 2 and 4 (*p* < 0.05). These results suggest that ECL7 reduced the richness and diversity of the bacterial community compared to the control group.

The PCoA results based on the ASVs indicated no significant differences in microbial compositions between the control and ECL7-treated groups on the 1st, 2nd, and 4th days (Figures 5C–E). Supplementary Figure S1 shows that the microbial community of the control group was clustered on the 1st, 2nd, and 4th days. However, the summary plots of the ECL7 treatment for days 1, 2, and 4 (Figure 5F) showed that samples from days 1 and 2 clustered together, whereas samples from the ECL7-treated group on day 4 clustered separately from the control group. This suggests that prolonged ECL7 infection significantly altered the gut microbiota balance (*p* < 0.05).

### 3.6 Gut microbiota changes in silkworms after ECL7 infection

**Figure 6:**
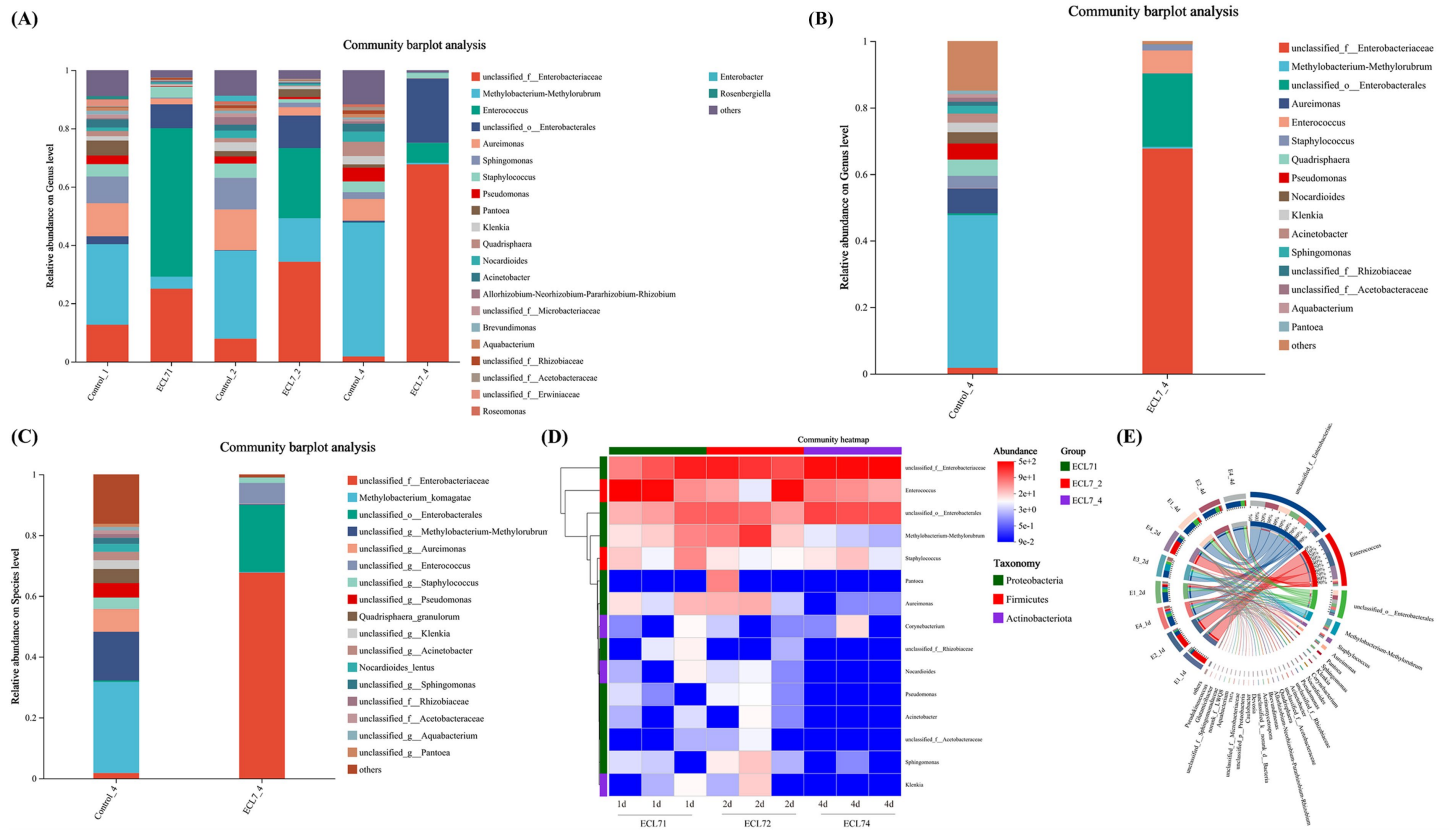
Effects of ECL7 infection on midgut community structures. **(A)** Relative abundance of the microbiota community in control and ECL7-infected groups at the genus level on the 1st, 2nd, and 4th days. **(B,C)** Relative abundance of the microbiota community in ECL7-infected groups at the genus and species levels on the 4th days. **(D)** Heatmap displaying major taxa in control and ECL7-infected groups. **(E)** Circle plots showing microbiota community abundance after ECL7 infection.

The microbial community composition and abundance following ECL7 treatment were analyzed. At the genus level, the primary changes observed included increases in the proportions of unclassified_f_*Enterobacteriaceae*, unclassified_o_*Enterobacterales*, and *Enterococcus*, compared to the control, with the exception of chloroplasts and mitochondria. In the ECL7 infected group, on the 1st, 2nd, and 4th days, the relative abundances of unclassified_ f_*Enterobacteriaceae* were 24.98, 34.27, and 67.70%, respectively, whereas those of unclassified_o_*Enterobacterales* were 8.21, 11.16, and 22.03%, respectively. Conversely, the relative abundance of *Enterococcus* decreased from 50.93 to 6.88% (Figure 6A). At the genus and species levels, the microbial community composition in the ECL7 group was altered, with unclassified_f_*Enterobacteriaceae* and unclassified_o_*Enterobacterales* emerging as the dominant species in the silkworm gut by day 4 (Figures 6B,C). Furthermore, a clustered heatmap illustrating the relative abundance of the dominant genera (top 15) and a circos plot of larval guts from the ECL7-treated group revealed that the proportions of unclassified_f_*Enterobacteriaceae* and unclassified_o_*Enterobacterales* in the gut microbiota gradually increased with prolonged treatment (Figures 6D,E). These results indicate that ECL7 significantly alters the intestinal microecology of silkworms.

### 3.7 Transcriptome analysis of DEGs and enrichment analyses

**Figure 7:**
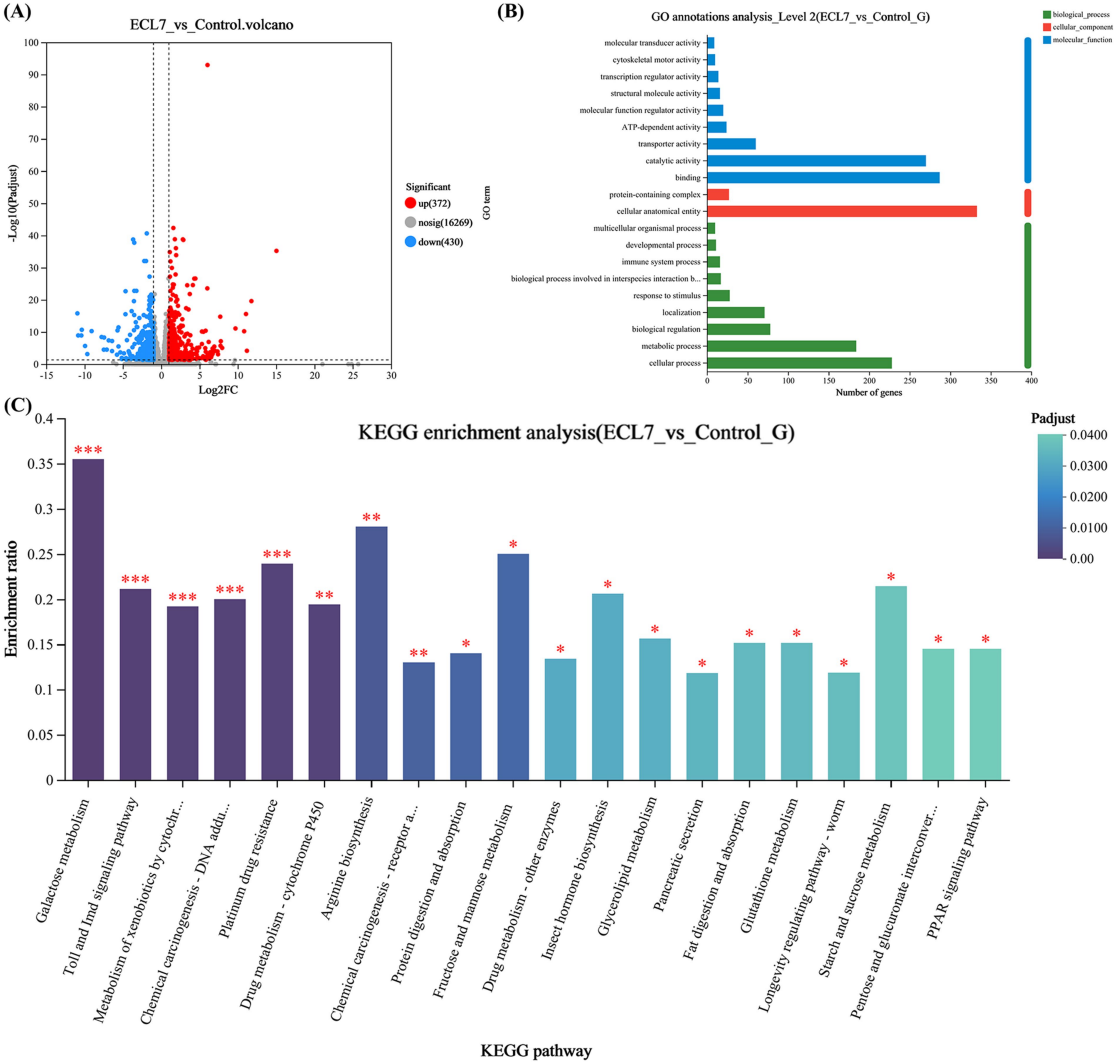
Transcriptomic analyses of the DEGs in the silkworm intestine 4 days after ECL7 infection. **(A)** Volcano plot of DEGs in the silkworm intestine. Red dots represent significantly upregulated DEGs, blue dots represent significantly downregulated DEGs, and gray dots represent non-significantly different DEGs. **(B)** GO annotation analysis; X-axis represents the GO terms, whereas Y-axis represents the enrichment ratio. **(C)** KEGG enrichment analysis; X-axis represents pathway classification, whereas Y-axis represents the enrichment ratio. The color indicates the significance of enrichment (corrected *p*-value), with the color gradient reflecting the degree of the *p*-value. *p < 0.05, **p < 0.01, ***p < 0.001.

Approximately 89.37 and 90.73% of clean reads from the control and ECL7 – treated groups were mapped to the reference genome. A total of 802 DEGs were identified, with 372 (46.4%) upregulated and 430 (53.6%) downregulated compared to the control (Figure 7A). DEGs were annotated in the GO database: 657 in biological processes (immune, cellular, metabolic, etc.), 724 in molecular functions (binding, catalytic, etc.), and 360 in cellular components (Figure 7B).

KEGG enrichment analysis of DEGs (422 mapped, 52.6%) showed the top 20 pathways (Figure 7C). KEGG enrichment analysis showed significant differences in the Toll and IMD pathways, galactose metabolism (*p* < 0.05), drug metabolism, arginine biosynthesis (*p* < 0.05), xenobiotic degradation and metabolism, protein degradation and uptake, glutathione metabolism, and starch and sucrose metabolism pathways (*p* < 0.05). Overall, the GO annotation and KEGG enrichment analyses indicated that the DEGs were closely associated with immune pathways, xenobiotic degradation and metabolism, amino acid metabolism, and carbohydrate and lipid metabolism in the silkworm intestine.

### 3.8 Response mechanism of the silkworm intestine after ECL7 infection

**Figure 8:**
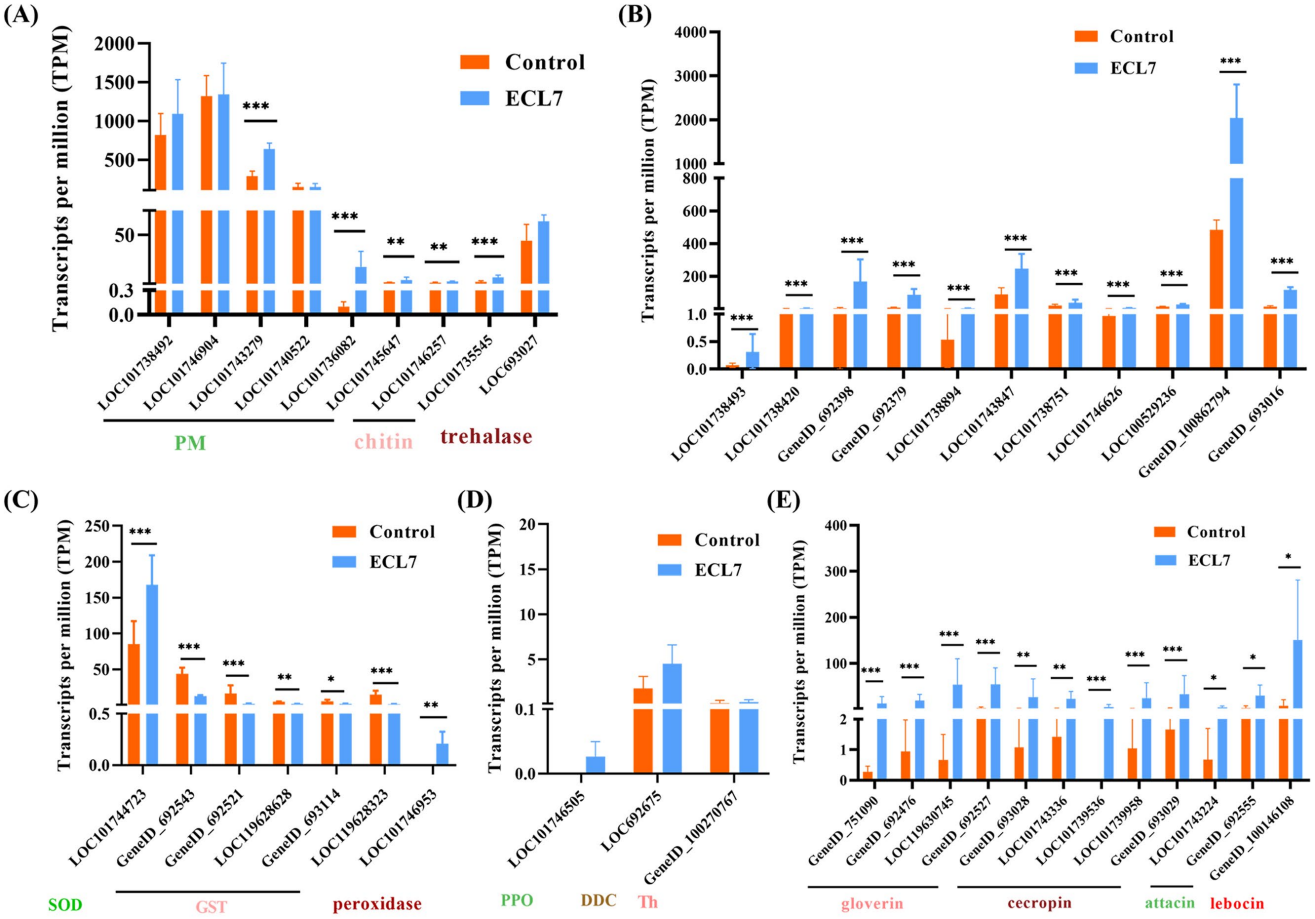
Effects of ECL7 infection on DEG transcripts. **(A)** DEGs associated with PM-related, chitin-related enzyme, and trehalase genes. **(B)** DEGs related to the IMD and TOll pathways. **(C)** DEGs associated with peroxidase. **(D)** DEGs related to melanization immunity. **(E)** DEGs related to antimicrobial peptides. Error bars indicate the mean ± SD (*n* = 3). *p < 0.05, **p < 0.01, ***p < 0.001.

The DEGs associated with PM, immune signaling pathways, oxidative stress, melanization immunity, and antimicrobial peptides (AMPs) were analyzed to elucidate the response mechanisms of the silkworm midgut following ECL7 infection. Genes encoding PM-related proteins (e.g., *LOC101743279* and *LOC101736082*) and chitin-related enzymes (e.g., *LOC101746257*, and *LOC101735545*) were significantly upregulated (*p* < 0.05). The gene encoding trehalase (*LOC693027*) showed higher expression than the control, though not significantly (*p* > 0.05, Figure 8A). In the midgut’s Toll and IMD immune pathways, genes including *LOC101738493*, *LOC101738420*, *GeneID_692398*, *GeneID_692379*, *LOC101738894*, *LOC101743847*, *LOC101738751*, *LOC101746626*, *LOC100529236*, *GeneID_100862794*, and *GeneID_693016*, were significantly upregulated (*p* < 0.05, Figure 8B), whereas glutathione-S-transferase-related genes, such as *GeneID_692543*, *GeneID_692521*, and *LOC119628323*, were significantly downregulated (*p* < 0.05). Conversely, genes encoding superoxide dismutase (SOD) and peroxidase (*LOC101744723*, *LOC101746953*) were significantly upregulated (*p* < 0.05, Figure 8C). Regarding melanization immunity, the expression levels of *BmDDC*, *BmTH*, and *BmPPO* (*LOC101746505*, *LOC692675*, and *GeneID_100270767*) were higher compared to the control group, although the differences were not significant (*p* > 0.05, Figure 8D). Additionally, the expression of genes encoding AMPs, including gloverin-encoding genes (*GeneID_751090*, *GeneID_692476*, *LOC119630745*, and *GeneID_692527*), cecropin-encoding (*GeneID_693028*, *LOC101743336*, *LOC101739536*, *LOC101739958*, and *GeneID_693029*), and attacin-encoding (*LOC101743224*, *GeneID_692555*) were all significantly upregulated (*p* < 0.05, Figure 8E).

To validate the RNA-seq data, 14 DEGs were randomly selected for qRT-PCR analysis, and the results showed trends of upregulation and downregulation consistent with the RNA-seq findings (Supplementary Figure S2).

## 4 Discussion

Currently, an increasing number of studies have shown that silkworms are vulnerable to attacks by pathogenic bacteria during sericultural processes, which may lead to their death (Wang et al., 2022). Pathogens present in deceased silkworms are released into the air and disseminated through aerosols, infecting healthy silkworms and causing significant economic losses to the silkworm industry (Saad et al., 2019). *E. cancerogenus*, a member of the ECC, is recognized as a pathogenic bacterium with implications for both human health and biological pest control (Çelik and Sevim, 2022). However, there is a lack of studies examining the pathogenicity of this bacterium in silkworms. Therefore, the present study aimed to investigate the potential pathogenic mechanisms of *E. cancerogenus* in the silkworm intestinal tract. The findings aim to improve the prevention of bacterial diseases caused by this bacterium and to promote its potential application in biological pest control within the agricultural field.

Due to the high similarity in pathogenicity and drug resistance among members of the ECC, accurately classifying these organisms remains challenging, even when using gene sequencing techniques. Consequently, many studies classify these bacteria collectively as ECC (Mustafa et al., 2020; Nishida et al., 2020; Suzuki et al., 2024). In this study, a phylogenetic tree was constructed using the neighbor-joining method in conjunction with ANI, which identified strain ECL7 as *E. cancerogenus*, a member of the ECC. Subsequently, whole-genome sequencing of ECL7 confirmed the presence of a plasmid, pC45-p2.

Bacterial virulence factors play a crucial role in enhancing the pathogenicity of microorganisms, enabling them to infect hosts, survive and reproduce within the host, and cause damage (Leitão, 2020). In our study, analysis using the VFDB revealed that ECL7 contains numerous virulence factors. For example, *fimA* enhances bacterial adhesion and promotes adaptability to the host environment, thereby increasing pathogenicity (Kato et al., 2007). Biofilm formation is a critical survival strategy for bacteria, aiding survival in adverse environments and further enhancing pathogenicity. *CsgA* promotes biofilm formation, allowing bacteria to persist within the host, facilitating sustained virulence factor release, and exhibiting a synergistic effect with other pathogenic genes, thereby increasing the overall pathogenic capacity of the bacteria (Uhlich et al., 2009; El Hag et al., 2017; Iswarya Jaisankar et al., 2020). Additionally, genes encoding ECP proteins enhance bacterial adhesion (Rossez et al., 2014). Furthermore, studies indicate that the type VI secretion system (T6SS) in *E. cancerogenus* can damage *Helicoverpa armigera* midgut cells, evading defenses and killing the host (Pan et al., 2024). The T6SS employs a contact – reliant secretion mechanism, through which it translocates a wide spectrum of lethal effector molecules into the neighboring prokaryotic or eukaryotic cells. This functional characteristic endows bacteria with a competitive edge in contact – mediated interactions, particularly in the context of bacterium – host relationships and inter – bacterial competitions (Russell et al., 2014). ECL7 possesses 13 core genes (*tssA–M*) essential for the T6SS, which are similar to those found in other bacteria (Cherrak et al., 2019; Navarro-Garcia et al., 2019). This similarity suggests that ECL7 also exhibits horizontal gene transfer potential, as reported for *E. cloacae* by Kenji Watanabe et al. (Watanabe and Sato, 1998). However, the role of horizontal gene transfer in ECL7 and its impact on the pathogenicity of ECL7 in *Bombyx mori* need to be further investigated in the future. This study indicates that after ECL7 infection, silkworms grow more slowly. As the infection duration prolonged, the mortality rate among silkworms gradually increased, suggesting that ECL7 infection in silkworms is chronic. Taken together, the pathogenicity of ECL7 in silkworms may be attributed to the combined effects of multiple virulence factors released by ECL7. These factors may facilitate the formation of biofilms and other structures as well as the transfer of ECL7 virulence genes to other microorganisms within the silkworm midgut. Consequently, other bacterial species may acquire ECL7 pathogenic genes, enabling their spread and reproduction in the midgut, thereby delaying silkworm growth, development and leads to silkworm death. However, the role of virulence genes in ECL7, such as *fimA*, *T6SS* and other virulence genes, will be the direction of further investigation in the future.

Entomopathogenic bacteria infect the intestinal tract of insects through ingestion, where they proliferate and induce disease. These bacteria release toxins and other virulence factors targeting midgut cells, damaging the epithelial barrier, entering the main cavity, and killing the insect host (Jurat-Fuentes and Jackson, 2012). PM is a composite membrane structure made of chitin and protein, and is the first line of immune defense, limiting direct contact between bacterial toxins and the midgut epithelium (Hegedus et al., 2009). In their study, they observed that the silkworms died gradually after 96 h of continuous inoculation (Li et al., 2020; Li et al., 2021; Zhu et al., 2023). In our study, although the silkworm larvae started to exhibit mortality from the first day of ECL7 infection, histopathological examinations revealed that in the early days post – infection (including day 1 and day 2), the PM and intestinal epithelial cells of the silkworms were not significantly damaged. However, by day 4, severe damage was observed. This early – stage mortality might be attributed to a rapid and acute physiological response. During the initial phase of infection, some larvae that were more susceptible to the pathogen died on the first day. In contrast, as the infection progressed to the late stage, the virulence factors of ECL7 enabled the bacterium to gradually adapt to the intestinal microenvironment of the silkworm. Subsequently, ECL7 proliferated extensively, penetrated into the main body lumen of the silkworm, and inflicted damage on the PM and intestinal epithelial cells, thereby increasing the silkworm’s vulnerability to this pathogen. The alterations in the silkworm were a cumulative process. In the early stages of infection, the changes in gene expression and tissue structure were so subtle that they could not be detected by histopathology. As the infection advanced, these minute changes gradually accumulated and reached a level where they could be detected by histopathology. Consequently, by day four, significant damage to the PM and intestinal epithelium of the silkworms was observed.

Insect gut microorganisms not only help obtain nutrients but also detoxify pesticides, which are vital for insect health (Pietri et al., 2018). *Enterococcus* bacteria produce bacteriocins affecting gut microbiota (Gomes et al., 2020). In this study, to accurately analyze the community composition of the gut microbiota in silkworms, we removed the chloroplast and mitochondrial sequences. After ECL7 infection, the diversity of the silkworm gut microbiota was notably altered. The relative abundances of unclassified_f_*Enterobacteriaceae* and unclassified_o_*Enterobacterales* increased steadily with the elongation of the infection period. In contrast, the relative abundance of *Enterococcus* decreased. It is clear that this increase in relative abundance is a direct consequence of ECL7 infection. This result strongly implies that ECL7 has taken over the ecological niche of *Enterococci* and become the dominant species in the silkworm intestine. This shift ultimately alters the intestinal microecology of silkworms. This result may be related to the virulence genes in ECL7. For instance, T6SS is essential for bacteria to adapt to and colonize various environments, playing a key role in interbacterial competition, adherence, and colonization of host cells (Mustafa et al., 2020; Gallegos-Monterrosa and Coulthurst, 2021; Yin et al., 2024). Furthermore, we identified *narG* in the ECL7 genome, which promotes bacterial survival in anaerobic environments (Gregory et al., 2003). Changes in the gut microecology of silkworms on days 1, 2, and 4 of infection were consistent with changes in mortality and histopathological trends, and the PM and gut epithelial cells of silkworms were severely damaged by day 4 of ECL7 infection, which may increase their susceptibility to this pathogen. Studies have shown that disrupted PM increases host susceptibility to pathogens and that the use of chlorantraniliprole disrupts the PM and immune system of the silkworm, leading to intestinal microecological imbalances and increasing the risk of pathogenic bacterial infections (Zhu et al., 2023). Our findings suggest that the invasion of the *Bombyx mori* gut by ECL7 likely involves intricate ecological competition and succession processes.

Previous evidence indicates that AMPs, immune responses, and gut microorganisms are closely interconnected. Mucin forms a physical barrier to prevent the entry of bacteria and viruses and plays an important role in maintaining the diversity and stability of gut microbiota. Chitin deacetylase helps to maintain the integrity of the PM around the intestine and enhance the host’s resistance to pathogens (Tang et al., 2024). RNA-seq analysis on the 4th day revealed the expression levels of DEGs in the midgut PM of silkworms were significantly upregulated after ECL7 infection. Such as the expression levels of genes encoding mucin (*LOC101743279*) and chitin deacetylase (*LOC101746257*), which are associated with PM integrity, were significantly elevated. This indicates that on the fourth day after the silkworms are infected with ECL7, the PM is damaged, which not only increases the risk of ECL7 infecting the silkworm gut but also alters the ecological structure of the silkworm gut microbiota. *Francisella tularensis* establishes host resistance by forming a symbiotic relationship with silkworms, suggesting that the bacterium enhances its survival by modulating the host’s immune response (Suzuki et al., 2016). Furthermore, we identified *acrB*, which encodes an efflux pump that enhances bacterial resistance to AMPs (Kobylka et al., 2020). The Toll and IMD pathways are core signaling pathways in the insect immune system. The abundance and diversity of intestinal microorganisms can influence IMD pathway expression (Barletta et al., 2017). The Toll pathway complements IMD to enhance intestinal immune defense (Alejandro et al., 2022). In *Drosophila*, microbiota imbalance can cause overactivation of the IMD pathway, resulting in chronic inflammation and other immune-related diseases (Buchon et al., 2009). Feeding silkworm-specific microbes can boost AMP synthesis and the immune response (Romoli et al., 2017; Wu et al., 2010). In this study, RNA-seq results indicated that the genes related to Toll and IMD pathways and AMPs were upregulated in the silkworm midgut on the 4th day after ECL7 infection. Our research speculates that though the Toll and IMD pathways and AMPs are activated after ECL7 infection, the pathogenicity of ECL7 in silkworms may be further augmented due to its ability to evade host immunity.

Additionally, the expression levels of certain oxidative stress-related enzymes, such as those encoding SOD and peroxidases, were upregulated. This may be because when silkworms are infected with bacteria, they produce not only AMPs but also reactive oxygen species (ROS) to combat invading pathogens (Zhang et al., 2015). However, excessive ROS can also cause damage to host cells. Consequently, cells must clear excess ROS to mitigate tissue damage and maintain the normal functioning of the immune system (Vatansever et al., 2013). In addition, the silkworm host may die due to the collapse of energy metabolism. Moreover, transcriptome analysis revealed that ECL7 infection affects multiple pathways related to energy metabolism, including amino acid, glucose, and lipid metabolism. Notably, the expression of *BmDDC*, *BmTH*, and *BmPPO*, which are key genes involved in melanization immunity, did not change significantly. This suggests that melanization is not part of the immune response induced by ECL7.

## 5 Conclusion

In summary, our results highlight the impact of enterogenic *E. cancerogenus* on the intestinal microbiome and immune response of silkworms. Our study indicates that multiple virulence genes in ECL7 compromise the PM and intestinal epithelial cells of silkworms, affecting the health of silkworm larvae. Furthermore, ECL7 induces an imbalance in the intestinal microecology of silkworms, leading to the upregulation of genes associated with oxidative stress, immune signaling pathways, and AMPs. Additionally, ECL7 affects various biological processes and metabolic pathways in silkworms. This study provides new insights into the interaction between *E. cancerogenus* and insect hosts and highlights the potential application of *E. cancerogenus* in biological pest control.

## Data Availability

The datasets presented in this study can be found in online repositories. The names of the repository/repositories and accession number(s) can be found at: https://www.ncbi.nlm.nih.gov/, PRJNA1171164; PRJNA1172178; PRJNA1172358.

## Footnotes

^1^ http://soap.genomics.org.cn/

^2^ https://www.megasoftware.net/

^3^ https://github.com/smaegol/PlasFlow

^4^ https://blast.ncbi.nlm.nih.gov/Blast.cgi

^5^ https://ccb-microbe.cs.uni-saarland.de/plsdb/

^6^ http://topaz.gatech.edu/GeneMark

^7^ http://ccb.jhu.edu/software/glimmer/index.shtml

^8^ http://www.mgc.ac.cn/VFs/main.htm

^9^ https://benjjneb.github.io/dada2/tutorial.html

^10^ https://qiime2.org

^11^ https://github.com/jstjohn/SeqPrep

^12^ https://github.com/najoshi/sickle

^13^ https://metazoa.ensembl.org/Bombyx_mori_gca014905235v2/Info/Index

^14^ http://tophat.cbcb.umd.edu/

^15^ http://ccb.jhu.edu/software/stringtie/

^16^ https://github.com/tanghaibao/GOatools
