# Supplementary material for: Pathogenicity and host-interacting mechanisms of enterogenic Enterobacter cancerogenus in silkworm
Source: Front Microbiol. 2025 Mar 26;16:1548808. doi: 10.3389/fmicb.2025.1548808 (PMC11979111; doi:10.3389/fmicb.2025.1548808)
Supplement: Supplementary file 1 [file Data_Sheet_1.docx]

Supplementary Material

# Supplementary Figures


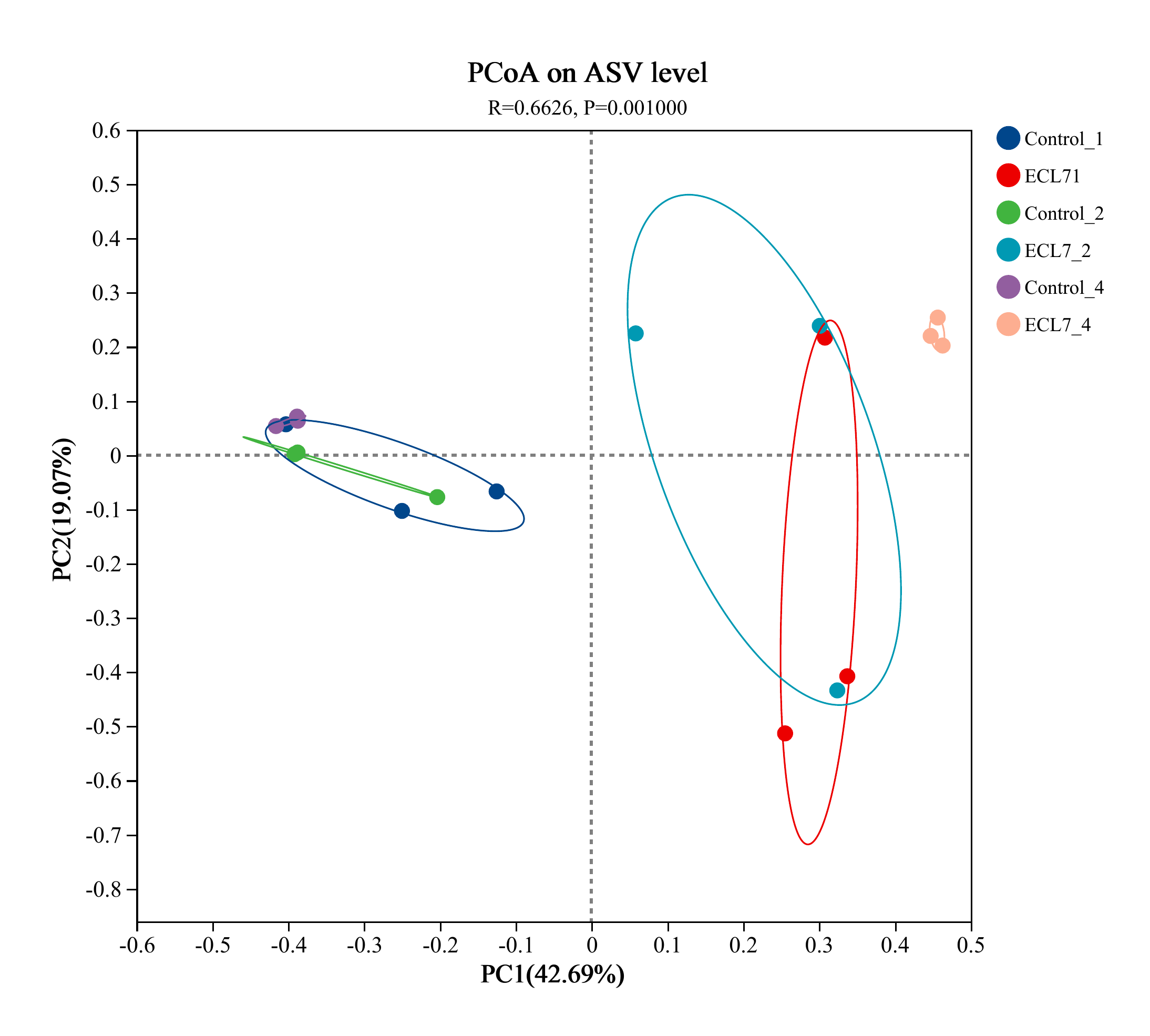


**Supplementary Figure S1** | Effects of ECL7 infection on midgut microbial diversity indices of all groups on days 1, 2, and 4.


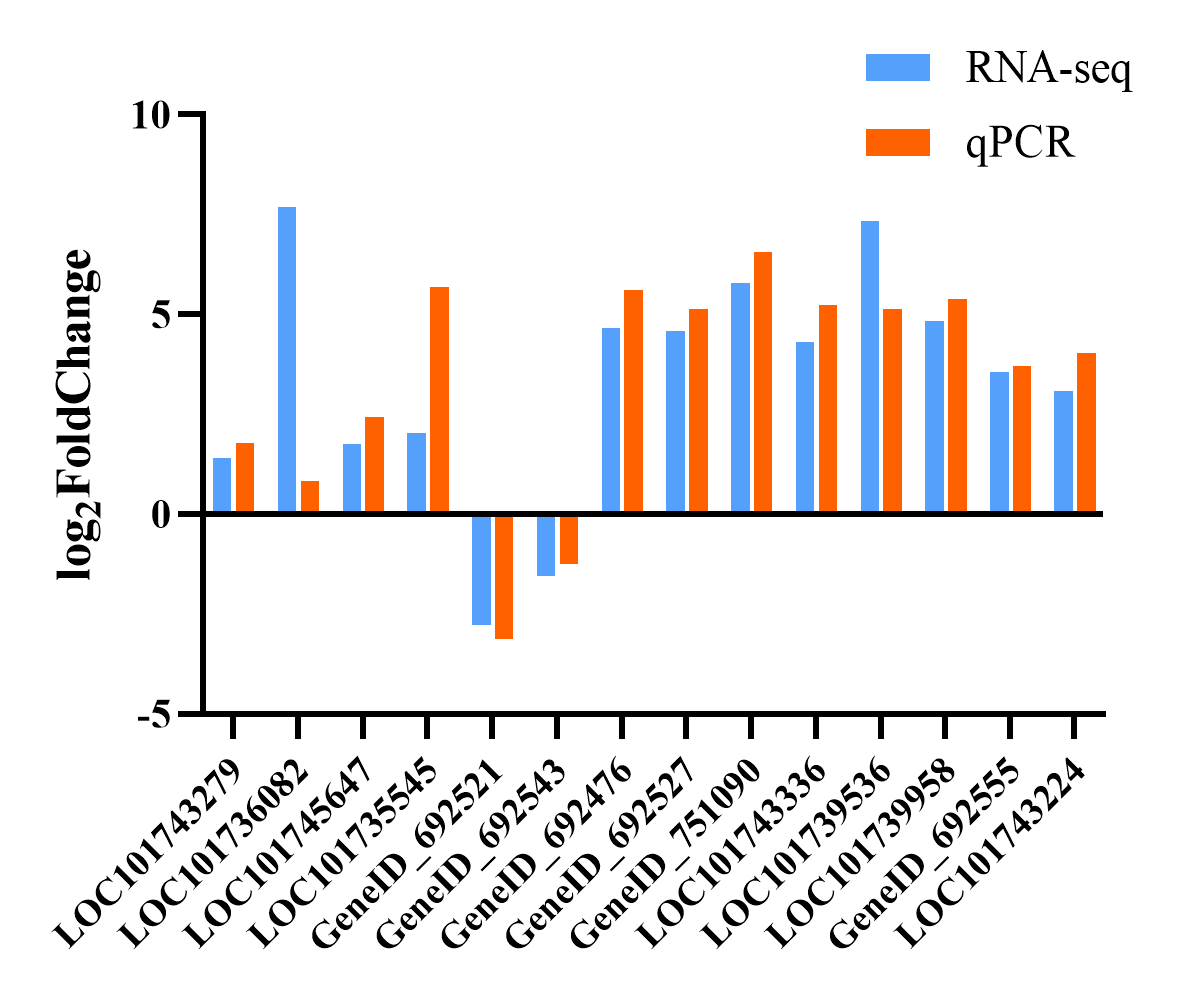


**Supplementary Figure S2.** **|** qRT-PCR verification of the transcriptome. The experiment was repeated thrice. The Y-axis represents the log_2_ fold change in gene expression. All transcriptional data were normalized to the actin expression level.

# Supplementary Tables

**Supplementary Table S1.** Real-time PCR primers used in this study.

| **Gene (total number)** | **Primers** | **Nucleotide sequence (5’-3’)** | **Product size (bp)** |
| --- | --- | --- | --- |
| *LOC101743279* | LOC101743279 F | GCGTCACGACAACGTATGAA | 111 |
|  | LOC101743279 R | TGAAATTGTCGCCGATGACC |  |
| *LOC101736082* | LOC101736082 F | GGTCAGCCAGACATACGCAAGG | 105 |
|  | LOC101736082 R | CGTGGTGGTCGTGGTTGTAGTG |  |
| *LOC101745647* | LOC101745647 F | CTCCAATTACAGCTGCACCA | 154 |
|  | LOC101745647 R | GTGGTTGTAACGGCAGGTG |  |
| *LOC101735545* | LOC101735545 F | CTGCCCAACCGCTTCTATTC | 179 |
|  | LOC101735545 R | CCCTGTTTCCGAGGTAGTGT |  |
| *GeneID_692521* | GeneID_692521 F | CCTGGAAGCCTCTGACATTG | 114 |
|  | GeneID_692521 R | GCCTCCAGTCCTTTCTCGTT |  |
| *GeneID_692543* | GeneID_692543 F | ATCGACCAGAACGTGGAGTT | 104 |
|  | GeneID_692543 R | TCCAGTTCCGCCTTCTTCTT |  |
| *GeneID_692476* | GeneID_692476 F | CTCCGGTGTGTGGGATCTT | 112 |
|  | GeneID_692476 R | ATGCCGGATCTCTGCTTGAA |  |
| *GeneID_692527* | GeneID_692527 F | GCGAACAAGAATGCACAAGC | 194 |
|  | GeneID_692527 R | CACCAATCATGGCGGATCTC |  |
| *GeneID_751090* | GeneID_751090 F | AACAAGTGGGAGGAGGGAAG | 172 |
|  | GeneID_751090 R | CCACCGTAGTTTGTACTGTCC |  |
| *LOC101743336* | LOC101743336 F | TTGAGCTTCGTCTTCGCGTT | 137 |
|  | LOC101743336 R | ATAGCTGGACCCGCTTTGAT |  |
| *LOC101739536* | LOC101739536 F | ATCCTTCGTCTTCGCTCTGG | 165 |
|  | LOC101739536 R | CCTATAGCTTTAGCCGAACCG |  |
| *LOC101739958* | LOC101739958 F | ATCCTTCGTCTTCGCTCTGG | 106 |
|  | LOC101739958 R | ACGGATGTTCCTGCCCATTT |  |
| *GeneID_692555* | GeneID_692555 F | CAGTGAACTCGGATGGAACC | 158 |
|  | GeneID_692555 R | GTGCCCGTTTACATTGTCCA |  |
| *LOC101743224* | LOC101743224 F | ATAACAACCACGACCTGAGC | 126 |
|  | LOC101743224 R | CGCCCACCTTCTGTTTGAA |  |
| *Actin 3* | Actin 3 F | CGGCTACTCGTTCACTAC | 147 |
|  | Actin 3 R | CCGTCGGGAAGTTCGTAAG |  |

**Supplementary Table S2.** Housekeeping genes used for constructing the phylogenetic tree of *E.* *cancerogenus* strain ECL7.

| **Sample Name** | **Hit ID** | **Hit species** | **Identity (%)** | **Coverage (%)** |
| --- | --- | --- | --- | --- |
| ECL7 | GCF_019047785.1 | *Enterobacter_cancerogenus* | 99.5 | 81.34 |
| ECL7 | GCF_001984825.2 | *Enterobacter_chengduensis* | 98.9 | 81.34 |
| ECL7 | GCF_900324475.1 | *Enterobacter_bugandensis* | 98.8 | 81.34 |
| ECL7 | GCF_003057745.1 | *Enterobacter_sp003057745* | 98.8 | 81.34 |
| ECL7 | GCF_001654845.1 | *Enterobacter_soli* | 98.7 | 81.34 |
| ECL7 | GCF_001598695.1 | *Klebsiella_oxytoca* | 97.3 | 81.34 |
| ECL7 | GCF_001888805.2 | *Enterobacter_D_sp001888805* | 96.9 | 81.34 |
| ECL7 | GCF_001277175.1 | *Cronobacter_universalis* | 96.6 | 81.34 |
| ECL7 | GCF_000696575.1 | *Siccibacter_colletis* | 96.2 | 81.34 |
| ECL7 | GCF_009711095.1 | *Escherichia_C_alba* | 94.4 | 89.16 |
| ECL7 | GCF_000599885.1 | *Erwinia_sp000599885* | 93.7 | 81.3 |
| ECL7 | GCF_000590885.1 | *Erwinia_mallotivora* | 93.6 | 81.3 |
| ECL7 | GCF_000404125.1 | *Erwinia_tracheiphila* | 93.5 | 81.3 |
| ECL7 | GCF_002291445.1 | *Brenneria_goodwinii* | 93.4 | 81.3 |
| ECL7 | GCF_004195115.1 | *Rahnella_sp004195115* | 93.3 | 81.3 |
| ECL7 | GCF_003332275.1 | *Edaphovirga_cremea* | 93.1 | 81.3 |
| ECL7 | GCF_000688655.1 | *Lonsdalea_quercina* | 92.9 | 81.3 |
| ECL7 | GCF_011752625.1 | *Pantoea_sp011752625* | 90.9 | 89.16 |
| ECL7 | GCF_018448935.1 | *Photorhabdus_noenieputensis* | 88.3 | 89.13 |

**Supplementary Table S3.** Prediction of virulence genes in *E. cancerogenus* strain ECL7.

| **Sample Name** | **VF category** | **Gene No.** |
| --- | --- | --- |
| ECL7 | Nutritional/Metabolic factor | 155 |
| ECL7 | Motility | 110 |
| ECL7 | Adherence | 107 |
| ECL7 | Immune modulation | 88 |
| ECL7 | Effector delivery system | 82 |
| ECL7 | Regulation | 53 |
| ECL7 | Biofilm | 39 |
| ECL7 | Exotoxin | 25 |
| ECL7 | Antimicrobial activity/Competitive advantage | 19 |
| ECL7 | Stress survival | 13 |
| ECL7 | Invasion | 5 |
| ECL7 | Others | 4 |
| ECL7 | Exoenzyme | 3 |
| ECL7 | Post-translational modification | 2 |
